# Teacher autonomy support and music learning engagement: conditional associations of self-efficacy and discrete achievement emotions among university music students

**DOI:** 10.3389/fpsyg.2026.1947710

**Published:** 2026-09-29

**Authors:** Honghua Xu, Wei Liu

**Affiliations:** 1 College of Music, Guizhou University, Guiyang, China; 2 Graduate School, José Rizal University, Mandaluyong, Philippines

**Keywords:** achievement emotions, music learning engagement, music learning self-efficacy, teacher autonomy support, university music students

## Abstract

**Objective:**

University music learning requires sustained engagement under demanding instructional and practice conditions. This study examined whether perceived teacher autonomy support, music learning self-efficacy, and discrete achievement emotions showed distinguishable associations with multidimensional engagement when considered simultaneously among Chinese undergraduate music students.

**Methods:**

Cross-sectional survey data were collected from 500 undergraduate music students enrolled at three universities in southern China. Participants reported on one current core music course and its primary instructor. Covariance-based structural equation modeling was used to evaluate the measurement and structural models, with music learning engagement specified as a higher-order construct comprising behavioral, cognitive, and agentic engagement. Indirect associations were estimated using 5,000 case-resampling bootstrap replications with 95% bias-corrected confidence intervals.

**Results:**

The measurement and structural models showed good fit. Perceived teacher autonomy support was positively associated with music learning self-efficacy and retained a small positive conditional association with engagement. Self-efficacy was positively associated with enjoyment and negatively associated with anxiety and boredom. When all variables were modeled simultaneously, enjoyment was positively associated with engagement and boredom was negatively associated with engagement, whereas anxiety and self-efficacy showed no statistically distinguishable direct associations with engagement. The theoretically ordered indirect associations through self-efficacy and enjoyment [*β* = 0.209, 95% CI (0.099, 0.359)] and through self-efficacy and boredom [*β* = 0.042, 95% CI (0.011, 0.087)] were statistically distinguishable from zero, whereas the corresponding anxiety pathway was not. The model explained 29.5% of the variance in engagement.

**Conclusion:**

Perceived teacher autonomy support, competence-related beliefs, and discrete achievement emotions were differently associated with music learning engagement. In particular, enjoyment and boredom retained distinct associations with engagement after the other constructs were considered simultaneously. These findings support examining instructional support, self-efficacy, and discrete emotional experiences together rather than treating them as interchangeable correlates of university music learning engagement.

## 1 Introduction

University music study requires sustained cognitive, emotional, and behavioral investment across coursework, individual practice, ensemble rehearsal, and performance preparation. Students must develop technical and interpretive competence while regulating practice, responding to evaluative feedback, and managing the emotional demands of instruction and assessment. Engagement is therefore central to music learning because students must sustain effort, apply appropriate learning strategies, and participate actively in instructional processes (Bonneville-Roussy and Evans, 2025). Recent research further indicates that music students’ learning experiences are associated not only with instructional content but also with perceived teacher support, competence beliefs, and course-related emotions (Herrera et al., 2021; Xu and Li, 2025; Zelenak, 2024). What remains unclear is whether perceived teacher autonomy support, competence-related beliefs, and discrete emotions show distinguishable associations with engagement in university music learning.

Among these instructional factors, teacher autonomy support is particularly relevant because it concerns how instructors support student initiative within demanding learning environments. Grounded in self-determination theory, autonomy-supportive teaching involves acknowledging students’ perspectives, providing meaningful rationales and appropriate opportunities for choice, encouraging initiative, and minimizing unnecessarily controlling practices (Reeve and Cheon, 2021). It should be distinguished from broader teacher support, which may also include emotional, instrumental, academic, and structural assistance; studies of broader teacher support are therefore used here only as contextual rather than conceptually equivalent evidence. Autonomy support does not imply permissiveness or reduced guidance but can coexist with clear expectations, informative feedback, and appropriate structure (Patzak and Zhang, 2025). University music learning provides a particularly informative setting for examining autonomy support because instruction is simultaneously expert-directed and student-dependent. Teachers must communicate technical and interpretive standards and provide corrective guidance, while students must increasingly regulate their own practice and make interpretive decisions. Across classroom and higher education settings, perceived autonomy-supportive teaching has been associated with stronger engagement and more adaptive motivational functioning (Lauermann and Berger, 2021; Zhao and Qin, 2021).

Chinese university music education provides a relevant context for examining autonomy-supportive teaching because students often learn within structured course and performance requirements while also being expected to develop increasing responsibility for practice and artistic decision making. This coexistence of strong instructional structure and growing expectations for learner autonomy makes the context particularly relevant for examining autonomy-supportive teaching. Recent research with Chinese university music students has similarly linked autonomy-supportive music teaching with collaborative learning, flow, and well-being (Xu and Li, 2025). This context therefore provides a useful setting for examining how instructional support, competence-related beliefs, and emotional experiences are associated with student engagement.

Two limitations in existing music education research are particularly relevant. First, research on music students’ emotions has focused heavily on music performance anxiety (Barros et al., 2022), although students also experience enjoyment and boredom during coursework, lessons, rehearsals, and practice. These emotions differ in valence and activation and may show distinct associations with learning behavior (Tze et al., 2022). Examining them simultaneously may therefore reveal patterns obscured by treating emotional experience as a single positive or negative dimension. Second, engagement is multidimensional, encompassing behavioral participation and persistence, cognitive investment and strategy use, and agentic contributions to instruction (Reeve et al., 2020; Wong and Liem, 2022). These dimensions are conceptually distinguishable but may also reflect a broader form of student investment in learning. Distinguishing enjoyment, anxiety, and boredom is therefore important because a general positive-versus-negative emotion framework may obscure whether these emotions relate differently to multidimensional engagement.

The present study examined these relationships among Chinese undergraduate music students reporting on one current core music course and its primary instructor. Specifically, it assessed whether perceived teacher autonomy support, music learning self-efficacy, and enjoyment, anxiety, and boredom showed distinguishable conditional associations with multidimensional engagement when modeled simultaneously. It also estimated theoretically ordered indirect associations through self-efficacy and each discrete emotion, while treating these cross-sectional estimates as model-dependent associations rather than evidence of temporal mediation.

## 2 Theoretical framework and hypotheses

### 2.1 Self-determination theory and an emotion-specific extension from control-value theory

The proposed framework uses self-determination theory as its primary foundation and control-value theory as a complementary account of achievement emotions. Self-determination theory proposes that students demonstrate more autonomous motivation and adaptive learning when instructional environments support their needs for autonomy, competence, and relatedness (Ryan and Deci, 2020). Autonomy-supportive teachers acknowledge students’ perspectives, provide meaningful rationales, encourage initiative, offer appropriate opportunities for choice, and minimize unnecessarily controlling practices (Reeve and Cheon, 2021). This perspective provides the primary basis for examining perceived teacher autonomy support in relation to students’ self-efficacy and engagement.

Control-value theory complements this account by explaining differences among achievement emotions. It proposes that enjoyment, anxiety, boredom, and other achievement emotions are associated with students’ appraisals of control over achievement activities and outcomes and the value attached to them (Pekrun, 2006; Pekrun and Perry, 2014). Music learning self-efficacy is relevant to, but not equivalent to, perceived control because it concerns students’ beliefs in their capability to manage learning demands successfully (Bandura, 1997). Self-efficacy therefore represents one competence-related belief rather than the full range of control appraisals specified by control-value theory, and the present study did not assess value appraisals.

The two perspectives are used selectively and complementarily. Self-determination theory provides the instructional basis for examining perceived autonomy support, whereas control-value theory provides an emotion-specific basis for distinguishing enjoyment, anxiety, and boredom and for considering how competence-related beliefs may correspond differently with these emotions. The proposed model therefore integrates selected propositions from both theories rather than constituting a complete test of either framework.

### 2.2 Teacher autonomy support and music learning self-efficacy

Music learning self-efficacy refers to students’ beliefs in their capability to organize and perform the actions required for successful music learning (Bandura, 1997). In university music courses, these beliefs include confidence in regulating practice, understanding instructional content, responding to feedback, resolving technical or interpretive problems, and meeting course expectations. Autonomy-supportive instruction may be associated with stronger self-efficacy because meaningful rationales, opportunities for initiative, and informational rather than controlling guidance can make demanding learning tasks appear more understandable and manageable. Research involving university students has likewise reported positive associations between perceived autonomy support and academic self-efficacy (Gutiérrez and Tomás, 2019).

*H1*: Perceived teacher autonomy support is positively associated with music learning self-efficacy.

### 2.3 Teacher autonomy support, self-efficacy, and achievement emotions

Achievement emotions arise in relation to achievement activities and outcomes. The present study focuses on enjoyment, anxiety, and boredom because they represent positive activating, negative activating, and negative deactivating emotions, respectively (Pekrun, 2006; Pekrun and Perry, 2014).

Students with stronger music learning self-efficacy may experience learning demands as more manageable and rewarding, which may correspond with greater enjoyment and lower anxiety. Stronger self-efficacy may also be associated with lower boredom by helping students regulate repetitive practice, recognize progress, and remain involved when tasks are demanding or routine. Because boredom can also arise from low task value, insufficient challenge, or excessive difficulty, this association should not be interpreted as universal across all learning conditions.

*H2a*: Music learning self-efficacy is positively associated with enjoyment.

*H2b*: Music learning self-efficacy is negatively associated with anxiety.

*H2c*: Music learning self-efficacy is negatively associated with boredom.

Perceived teacher autonomy support may also show emotion-specific associations beyond those represented by self-efficacy. Meaningful rationales and opportunities for initiative may correspond with greater enjoyment, reduced controlling pressure may correspond with lower anxiety, and participation in practice and interpretive decisions may correspond with lower boredom. These paths examine whether perceived autonomy support retains conditional associations with the three emotions after self-efficacy is considered. Research outside university music education has similarly linked broader forms of perceived teacher support with enjoyment and boredom, although broader teacher support is not conceptually equivalent to autonomy support (Zhao and Yang, 2022).

*H3a*: Perceived teacher autonomy support is positively associated with enjoyment.

*H3b*: Perceived teacher autonomy support is negatively associated with anxiety.

*H3c*: Perceived teacher autonomy support is negatively associated with boredom.

### 2.4 Achievement emotions and music learning engagement

Music learning engagement is represented as a multidimensional construct comprising behavioral participation and persistence, cognitive investment and strategy use, and agentic contributions to instruction (Reeve et al., 2020; Wong and Liem, 2022).

Achievement emotions may correspond with these forms of involvement by shaping attention, effort, strategy use, and action tendencies. Enjoyment is expected to be positively associated with engagement because it is consistent with persistence, concentration, exploration, and active participation, whereas boredom is expected to be negatively associated with engagement because it is associated with attentional withdrawal and reduced effort. Research in other educational contexts has similarly linked enjoyment with stronger engagement and boredom with weaker engagement (Zhao and Yang, 2022). Meta-analytic evidence also indicates contrasting associations of enjoyment and boredom with academic performance, although performance and engagement remain distinct outcomes (Camacho-Morles et al., 2021).

*H4a*: Enjoyment is positively associated with music learning engagement.

*H4b*: Boredom is negatively associated with music learning engagement.

The conditional association between anxiety and engagement is less certain. Anxiety may consume cognitive resources and encourage avoidance, but its activating quality may also accompany vigilance, preparation, and sustained effort. Its association with engagement may therefore depend on intensity, context, and the simultaneous presence of other emotions and self-efficacy. A directional hypothesis was consequently not imposed.

*RQ1*: What conditional association does anxiety show with music learning engagement when enjoyment, boredom, music learning self-efficacy, and perceived teacher autonomy support are modeled simultaneously?

### 2.5 Direct and sequential indirect associations

Perceived teacher autonomy support may remain conditionally associated with engagement after music learning self-efficacy and achievement emotions are modeled simultaneously. Such an association would indicate that the measured psychological variables do not fully account for the covariance between autonomy support and engagement. Prior research has likewise linked perceived autonomy support and broader forms of teacher support with student engagement (Gutiérrez and Tomás, 2019; Tao et al., 2022).

*H5*: Perceived teacher autonomy support is positively associated with music learning engagement when music learning self-efficacy and achievement emotions are modeled simultaneously.

Music learning self-efficacy may also retain a conditional association with engagement because students who feel more capable may be more willing to persist, use effective strategies, and participate actively. However, the direction and magnitude of this association after autonomy support and the three achievement emotions are modeled simultaneously remain theoretically uncertain. This path was therefore examined without a directional hypothesis.

*RQ2*: What conditional association does music learning self-efficacy show with music learning engagement when perceived teacher autonomy support, enjoyment, anxiety, and boredom are modeled simultaneously?

The model also estimates theoretically ordered sequential indirect associations linking perceived teacher autonomy support with engagement through self-efficacy and each achievement emotion. Based on the preceding hypotheses, positive sequential indirect associations were expected through enjoyment and boredom. Because self-efficacy, emotions, and engagement were measured at the same occasion, however, the proposed ordering is theoretical rather than temporal. These indirect estimates should therefore be interpreted as model-dependent decompositions of covariance rather than evidence of sequential mediation.

*H6a*: Perceived teacher autonomy support shows a positive sequential indirect association with music learning engagement through music learning self-efficacy and enjoyment.

*H6b*: Perceived teacher autonomy support shows a positive sequential indirect association with music learning engagement through music learning self-efficacy and boredom.

The anxiety sequence was examined without a directional hypothesis because the direction of the association between anxiety and engagement was theoretically uncertain.

*RQ3*: Does perceived teacher autonomy support show a theoretically ordered sequential indirect association with music learning engagement through music learning self-efficacy and anxiety?

Figure 1 presents the proposed model.

**Figure 1:**
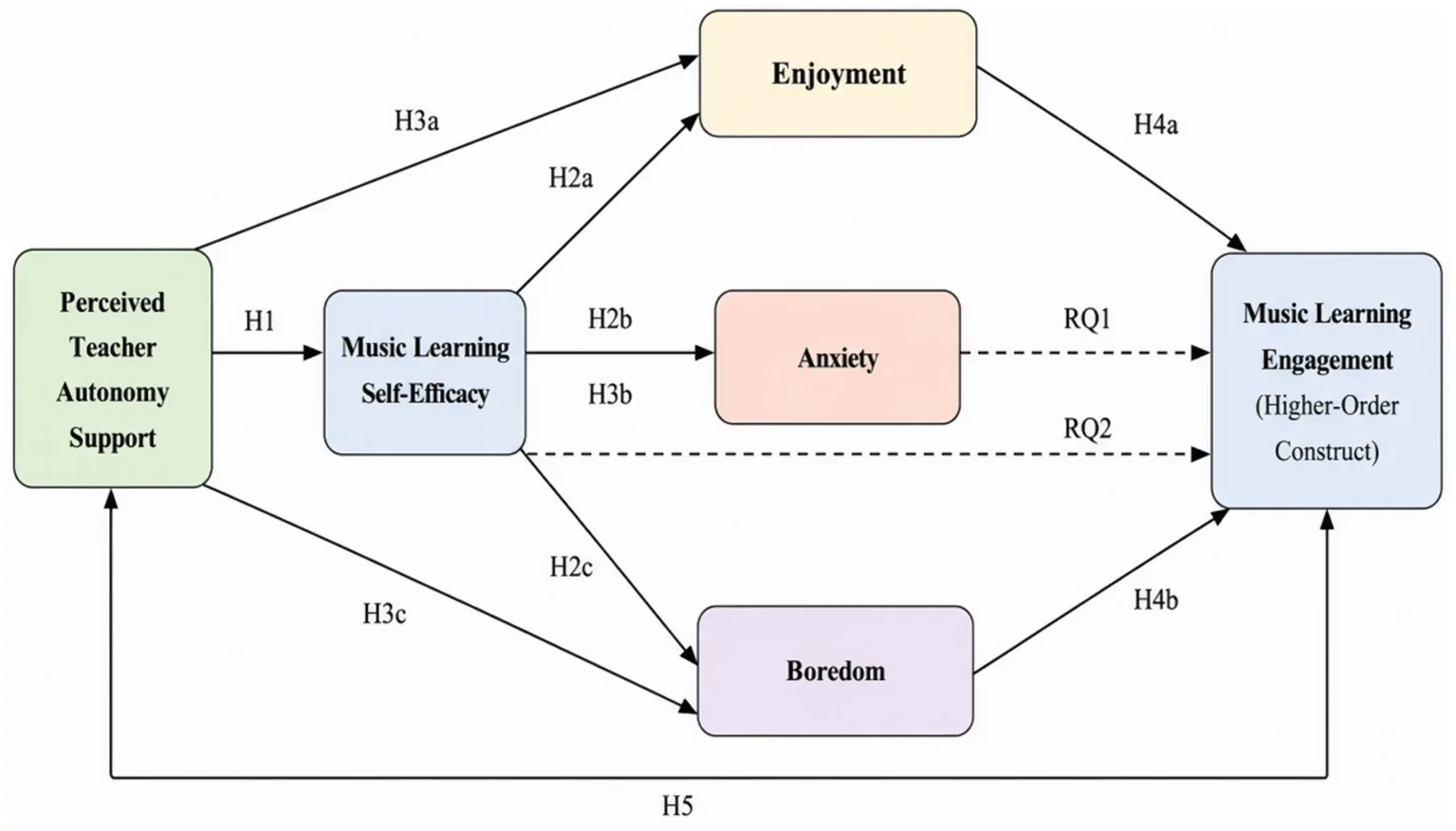
Proposed associations among teacher autonomy support, music learning self-efficacy, achievement emotions, and music learning engagement. Music learning engagement is modeled as a higher-order construct comprising behavioral, cognitive, and agentic engagement. Solid structural paths represent directional hypotheses, whereas dashed structural paths represent the exploratory associations addressed in RQ1 and RQ2. The theoretically ordered sequential indirect association through anxiety is examined under RQ3. All paths represent associations within the specified cross-sectional model rather than causal effects.

## 3 Method

### 3.1 Participants

The participants were 500 undergraduate music students recruited from three universities in southern China, including one comprehensive university, one normal university, and one specialized arts university. Convenience sampling was used at both the institutional and participant levels; the participating universities were accessible institutions that agreed to facilitate recruitment rather than institutions selected through probability sampling. Their differing institutional types provided some contextual variation in undergraduate music-education settings but should not be interpreted as statistically representative of Chinese universities.

Participants were recruited through an online survey administered via Wenjuanxing, with distribution of the survey link facilitated through contacts at the participating universities. Eligible participants were required to be currently enrolled undergraduate students, actively attending university courses during the data-collection period, and taking at least one core music course taught by an identifiable primary instructor. Institutional affiliation was not collected as an individual-level questionnaire variable and was not retained in the analytic dataset. Consequently, the final distribution of the 500 participants across the three universities cannot be reconstructed retrospectively.

The sample included 179 male students (35.8%) and 321 female students (64.2%). A total of 133 participants were first-year students (26.6%), 145 were second-year students (29.0%), 114 were third-year students (22.8%), and 108 were fourth-year students (21.6%). Regarding primary specialization, 117 students studied vocal music (23.4%), 157 studied instrumental music (31.4%), 96 studied musicology (19.2%), and 130 studied music education (26.0%). The demographic characteristics of the participants are presented in Table 1.

**Table 1:** Demographic characteristics of the participants.

Characteristic	Category	*n*	%
Gender	Male	179	35.8
	Female	321	64.2
Year of study	First year	133	26.6
	Second year	145	29.0
	Third year	114	22.8
	Fourth year	108	21.6
Primary specialization	Vocal music	117	23.4
	Instrumental music	157	31.4
	Musicology	96	19.2
	Music education	130	26.0

*N* = 500.

### 3.2 Data collection

Data collection commenced on 25 February 2026 and was completed in April 2026. The anonymous online questionnaire was administered through Wenjuanxing, a Chinese online survey platform. Before completing the questionnaire, participants were instructed to select one core music course in which they were currently enrolled and to use that course and its primary instructor as the reference for all subsequent responses. A total of 500 questionnaires were submitted. Because recruitment was conducted through distributed survey links rather than from an individually enumerated sampling frame, the exact number of eligible students who received or viewed the invitation was not recorded. Consequently, a conventional response rate could not be calculated. All scale and demographic items were configured as required-response fields, resulting in no item-level missing values.

No attention checks, response-time thresholds, straight-lining criteria, or multivariate-outlier exclusion rules had been prespecified before data collection. Consequently, post-hoc response-quality diagnostics were conducted using the available item-level data. These diagnostics examined long-string responding, within-person response variability across the 35 measurement items, and multivariate atypicality based on the eight construct composite scores. Completion-time metadata were not available in the analytic dataset and therefore could not be evaluated retrospectively. Because these diagnostics were conducted *post hoc* rather than specified in advance, they were treated as descriptive post-hoc diagnostics rather than as retrospective case-deletion rules. All 500 eligible cases were therefore retained in the primary analyses.

### 3.3 Instruments

The questionnaire included measures of perceived teacher autonomy support, music learning self-efficacy, achievement emotions, and music learning engagement. All item-selection, translation, and contextual-adaptation decisions were completed before examination of the main-study data and were guided by conceptual coverage of the intended constructs and the course-specific focus of the study rather than by observed factor loadings, model-fit statistics, or structural results. The original English materials were translated into Chinese by one bilingual member of the research team and independently back-translated into English by a second bilingual member. Both bilingual team members had backgrounds relevant to music education and educational research. The source, translated, and back-translated versions were then compared item by item to evaluate semantic equivalence, conceptual consistency, comprehensibility, and appropriateness for Chinese undergraduate music students. Any discrepancies were discussed and resolved before the adapted questionnaire was finalized for pilot testing. Contextual adaptation was subsequently undertaken to ensure that the administered items referred consistently to one current core music course, its primary instructor, and course-related learning or practice.

The extent of adaptation differed across the source measures. All six items of the short-form Learning Climate Questionnaire were retained, with wording contextualized to the focal course, instructor, and learning or practice activities. The music learning self-efficacy measure involved more substantial item reduction and contextual reconstruction because the present study focused on routine university course learning and practice rather than preparation for a specific public performance. For music learning engagement, behavioral, cognitive, and agentic engagement were retained, whereas emotional engagement was not included because learning enjoyment was assessed separately as an achievement emotion. The learning-related enjoyment, anxiety, and boredom items from the AEQ-S were contextualized to music-course learning and practice.

An item-level adaptation record was prepared to document the source construct and source-item content, the item administered in the present study, the type of modification, and the rationale for each adaptation decision; this comparison is provided in Supplementary Table S1. All measurement items were administered using a five-point Likert response format ranging from 1 (strongly disagree) to 5 (strongly agree). The five-point format was consistent with the original AEQ-S but represented a modification of the response formats used in the Learning Climate Questionnaire and the Self-Efficacy for Musical Learning measure. The selected engagement items were likewise administered using the common five-point format adopted for the present questionnaire. Because item selection, wording, and response-format modifications may affect psychometric functioning, the reliability and validity evidence reported in the present study pertains specifically to the adapted versions administered in this sample and should not be interpreted as establishing psychometric equivalence with the source instruments. Before the main data collection, the adapted questionnaire was pilot-tested with 30 undergraduate music students to examine preliminary internal consistency. Cronbach’s alpha coefficients across the study measures ranged from 0.825 to 0.927, indicating satisfactory preliminary reliability. No formal measurement-invariance comparison between the adapted and source instrument formats was conducted.

#### 3.3.1 Teacher autonomy support scale

Perceived teacher autonomy support was assessed using all six items of the short form of the Learning Climate Questionnaire (Williams and Deci, 1996). No item from the six-item short form was omitted. The wording was contextualized so that responses referred consistently to the focal music course, its primary instructor, and, where relevant, students’ learning or practice activities. A sample adapted item was, “In this course, the instructor gives me opportunities to choose how I learn or practice.” The source response format was replaced by the common five-point Likert format used throughout the present questionnaire. The six items were specified as indicators of a single perceived teacher autonomy support factor.

#### 3.3.2 Music learning self-efficacy scale

Music learning self-efficacy was assessed using five course-focused items informed by content represented in the Self-Efficacy for Musical Learning measure developed by Ritchie and Williamon (2011). The source measure was developed in relation to musical learning and performance preparation, whereas the present study focused specifically on routine university course learning and practice. The adaptation therefore involved both item reduction and substantial contextual reconstruction rather than direct translation alone.

The five administered items represented students’ confidence in mastering course-related musical knowledge or skills, completing difficult learning tasks, resolving problems encountered during learning or practice, meeting course requirements, and adjusting their approach and continuing to improve following initially ineffective learning or practice. A sample item was, “I am confident that I can master the musical knowledge or skills required in this course.” Content tied primarily to auditions, competitions, concerts, or other formal public-performance situations was not retained because it did not correspond to the course-learning focus of the present study. The source response format was replaced by the common five-point Likert format used throughout the present questionnaire. The five items were specified as indicators of a single music learning self-efficacy factor. Given the extent of item reduction and contextual reconstruction, the adapted five-item measure should not be interpreted as psychometrically equivalent to the original instrument.

#### 3.3.3 Achievement emotions scale

Music learning enjoyment, anxiety, and boredom were each assessed using four items adapted from the learning-related scales of the Achievement Emotions Questionnaire–Short (AEQ-S; Bieleke et al., 2021). The four-item structure of each of the three learning-related emotion scales was retained. Most modifications involved contextualizing item wording and instructions to the focal music course and related learning or practice, whereas two anxiety items involved broader wording changes concerning concentration difficulty and physiological tension. These modifications are documented at the item level in Supplementary Table S1.

The enjoyment items assessed positive feelings about learning, challenge, and progress. A sample item was, “I enjoy the challenge of learning the content of this course.” The anxiety items assessed nervousness, worry about managing course-learning demands, anxiety-related concentration difficulty, and physical tension or discomfort. A sample item was, “I worry about whether I can cope with all the learning or practice tasks in this course.” The boredom items assessed boredom with course content, mind wandering, postponement of boring tasks, and difficulty concentrating. A sample item was, “The learning content in this course is sometimes so boring that my mind wanders.” Participants were instructed to report emotions experienced during course learning and practice. For the enjoyment and anxiety items, the instructions further clarified that responses should refer to learning-related experiences rather than formal stage performances, competitions, or performances in front of an audience; the boredom instructions referred generally to learning and practice in the focal course. The original five-point AEQ-S response format was retained. Enjoyment, anxiety, and boredom were modeled as three separate latent factors.

#### 3.3.4 Music learning engagement scale

Music learning engagement was assessed using 12 items selected and contextually adapted from the multidimensional engagement measures reported by Reeve and Tseng (2011). The present study retained behavioral, cognitive, and agentic engagement, with four items representing each dimension. Emotional engagement was not included because positive emotional experience was assessed separately through music learning enjoyment, thereby reducing conceptual and item-content overlap between engagement and achievement emotion.

Behavioral engagement assessed attention, effort, task completion, and concentration during course learning and practice. A sample item was, “I work hard to complete the learning and practice tasks required in this course.” Cognitive engagement assessed connecting new material with prior knowledge, planning, monitoring understanding or mastery, and adjusting ineffective learning strategies. A sample item was, “If a learning or practice method does not help me understand or master the material, I change my approach.” Agentic engagement assessed asking questions, communicating learning preferences and interests, and making constructive suggestions about learning activities. A sample item was, “I tell the instructor how I prefer to learn or practice.” The selected items were contextualized to the focal music course and related learning or practice and were administered using the common five-point format adopted for the present questionnaire. Behavioral, cognitive, and agentic engagement were specified as three first-order factors loading on a second-order music learning engagement factor.

### 3.4 Data analysis

Data were analyzed using SPSS 26.0 and AMOS 23.0. SPSS was used to examine missing values, response distributions, descriptive statistics, internal consistency, Pearson correlations, potential common method bias, and post-hoc response-quality diagnostics. Because no response-quality exclusion criteria had been prespecified, long-string responding and within-person response variability were examined as descriptive diagnostics rather than as retrospective case-deletion criteria. Exploratory Tukey-fence criteria were used to flag unusually long identical-response sequences and unusually low within-person response variability, defined as values above Q3 + 1.5 × IQR for long-string responding and below Q1–1.5 × IQR for within-person response variability, respectively. Multivariate atypicality was additionally evaluated using Mahalanobis distance based on the eight construct composite scores, with *p* < 0.001 used as a conservative diagnostic threshold. Confirmatory factor analysis and structural equation modeling were conducted in AMOS using maximum likelihood estimation. The hypothesized measurement model distinguished eight first-order factors: teacher autonomy support, music learning self-efficacy, enjoyment, anxiety, boredom, behavioral engagement, cognitive engagement, and agentic engagement. The hypothesized model was compared with less differentiated competing models, and behavioral, cognitive, and agentic engagement were subsequently specified as dimensions of a second-order music learning engagement factor. Model fit was evaluated using the chi-square statistic, comparative fit index, Tucker–Lewis index, root mean square error of approximation, and standardized root mean square residual (Hu and Bentler, 1999; Kline, 2016). Internal consistency and convergent validity were assessed using Cronbach’s alpha, composite reliability, standardized factor loadings, and average variance extracted, while discriminant validity was examined using heterotrait–monotrait ratios and comparisons with less differentiated measurement models (Henseler et al., 2015). After the measurement model was established, the hypothesized structural model was estimated. Structural paths were specified from perceived teacher autonomy support to music learning self-efficacy, from self-efficacy to enjoyment, anxiety, and boredom, and from the three achievement emotions to music learning engagement. Additional direct paths were estimated from perceived teacher autonomy support to the three emotions and engagement and from self-efficacy to engagement. These paths were treated as conditional statistical associations within the specified model rather than as causal effects. Specific indirect associations were examined using 5,000 case-resampling bootstrap replications and 95% bias-corrected bootstrap confidence intervals. Harman’s single-factor analysis and a single-factor CFA model were used only as limited descriptive diagnostics of whether one general factor dominated the item covariance. Procedurally, participation was anonymous and voluntary, and no personally identifiable information was requested. These procedural features and statistical diagnostics were not treated as capable of eliminating common method variance. Because all focal constructs were measured through student self-report at the same measurement occasion, common method variance could not be ruled out. Given the cross-sectional design, all direct, indirect, and total relationships were interpreted as statistical associations rather than evidence of temporal or causal processes.

## 4 Results

### 4.1 Preliminary analyses

All 500 participants provided complete responses to the measurement items and demographic questions, and no missing-data treatment was required. All cases satisfied the study eligibility criteria. Because no response-quality exclusion criteria had been prespecified, post-hoc response-pattern diagnostics were conducted using the 35 measurement items. No participant exhibited complete straight-lining. The longest sequence of identical consecutive responses ranged from 2 to 11 items, and within-person response standard deviations ranged from 0.677 to 1.627. Using exploratory Tukey-fence criteria for unusually long identical-response sequences and unusually low within-person response variability, seven cases (1.4%) were flagged by at least one response-pattern indicator. These cases were not automatically excluded because the criteria were applied retrospectively and the flagged patterns did not uniformly indicate inattentive responding. Mahalanobis-distance screening based on the eight construct composite scores identified no multivariate outliers at *p* < 0.001. Completion-time metadata were unavailable in the analytic dataset and could not be evaluated retrospectively. Accordingly, all 500 eligible cases were retained for the primary analyses.

Composite-score skewness values ranged from −0.141 to 0.205, and kurtosis values ranged from −0.854 to −0.706, indicating no severe departures from univariate normality. Descriptive statistics and Pearson correlations among the principal study variables are presented in Table 2. Composite scores were used only for descriptive statistics, distributional assessment, and bivariate correlations and were not used to estimate the latent structural model.

**Table 2:** Descriptive statistics, distributional characteristics, and correlations among the principal study variables.

Variable	*M*	SD	Skewness	Kurtosis	1	2	3	4	5	6
1. Teacher autonomy support	3.16	0.94	−0.106	−0.722	1					
2. Music learning self-efficacy	3.10	0.92	−0.023	−0.854	0.506***	1				
3. Enjoyment	3.10	0.97	−0.127	−0.772	0.373***	0.617***	1			
4. Anxiety	2.81	0.93	0.177	−0.721	−0.233***	−0.232***	−0.121**	1		
5. Boredom	2.79	0.93	0.205	−0.706	−0.308***	−0.278***	−0.183***	0.381***	1	
6. Music learning engagement	3.09	0.79	−0.141	−0.750	0.284***	0.273***	0.335***	−0.158***	−0.298***	1

*N* = 500. Pearson correlations, means, standard deviations, skewness, and kurtosis were calculated using composite scores. **p < 0.01. ***p < 0.001.

As shown in Table 2, teacher autonomy support was positively correlated with music learning self-efficacy, enjoyment, and music learning engagement and negatively correlated with anxiety and boredom. Music learning self-efficacy was positively correlated with enjoyment and engagement and negatively correlated with anxiety and boredom. Enjoyment was positively correlated with engagement, whereas anxiety and boredom were negatively correlated with engagement. Anxiety and boredom were positively correlated with each other.

### 4.2 Measurement model and construct validity

A confirmatory factor analysis was conducted to examine the distinctiveness of teacher autonomy support, music learning self-efficacy, enjoyment, anxiety, boredom, behavioral engagement, cognitive engagement, and agentic engagement. The hypothesized eight-factor model demonstrated good fit to the data, *χ*^2^(532) = 803.31, *p* < 0.001, CFI = 0.959, TLI = 0.954, RMSEA = 0.032, 90% CI [0.027, 0.036], and SRMR = 0.042. In practical terms, these fit indices indicated that the proposed eight-construct measurement structure represented the observed item relationships well.

The hypothesized model was compared with five less differentiated alternatives. These models combined the three engagement dimensions, combined the three achievement emotions, combined self-efficacy with enjoyment, combined both the engagement dimensions and achievement emotions, or represented all items using a single general factor. As reported in Table 3, all five alternative models demonstrated poorer fit than the hypothesized eight-factor model. Although the self-efficacy–enjoyment-combined model fit better than the other alternative models, it remained inferior to the hypothesized model. The deterioration in fit was statistically distinguishable, Δ*χ*^2^(7) = 159.03, *p* < 0.001. This comparison indicated that self-efficacy and enjoyment were better represented as related but distinct constructs rather than as a single common factor.

**Table 3:** Comparison of competing measurement models.

Model	*χ* ^2^	df	CFI	TLI	RMSEA	SRMR
Eight-factor model	803.31	532	0.959	0.954	0.032	0.042
Engagement-combined model	1265.55	545	0.891	0.881	0.051	0.051
Emotion-combined model	1893.39	545	0.796	0.777	0.070	0.103
Self-efficacy–enjoyment-combined model	962.34	539	0.936	0.929	0.040	0.047
Four-factor model	2343.42	554	0.729	0.709	0.080	0.107
Single-factor model	4032.76	560	0.475	0.442	0.111	0.119

In the engagement-combined model, behavioral, cognitive, and agentic engagement were represented by one factor. In the emotion-combined model, enjoyment, anxiety, and boredom were represented by one achievement-emotion factor. The self-efficacy–enjoyment-combined model represented music learning self-efficacy and enjoyment using one factor. The four-factor model combined both the engagement dimensions and the achievement emotions.

Reliability and convergent validity results are presented in Table 4. Cronbach’s alpha coefficients ranged from 0.758 to 0.868, composite reliability values ranged from 0.756 to 0.868, and standardized factor loadings ranged from 0.553 to 0.784.

**Table 4:** Reliability and convergent validity of the study constructs.

Construct	Items	Cronbach’s α	CR	AVE	Standardized loading range
Teacher autonomy support	6	0.868	0.868	0.524	0.682–0.745
Music learning self-efficacy	5	0.809	0.806	0.456	0.584–0.759
Behavioral engagement	4	0.805	0.805	0.509	0.657–0.746
Cognitive engagement	4	0.811	0.811	0.517	0.677–0.740
Agentic engagement	4	0.805	0.805	0.510	0.612–0.784
Enjoyment	4	0.815	0.816	0.526	0.675–0.772
Anxiety	4	0.758	0.756	0.439	0.591–0.742
Boredom	4	0.769	0.768	0.456	0.553–0.729

CR, composite reliability; AVE, average variance extracted.

As shown in Table 4, the AVE values exceeded 0.50 for perceived teacher autonomy support, behavioral engagement, cognitive engagement, agentic engagement, and enjoyment. In contrast, the AVE values for music learning self-efficacy, anxiety, and boredom were 0.456, 0.439, and 0.456, respectively, falling below the conventional 0.50 benchmark. Composite reliability nevertheless exceeded 0.70 for all three constructs, their indicators loaded significantly on the intended factors, all HTMT ratios remained below 0.85, and the competing measurement models supported differentiation among the constructs. These results provide qualified support for retaining the prespecified measurement structure within the present sample. However, the below-0.50 AVE values indicate comparatively modest convergent validity for self-efficacy, anxiety, and boredom, and structural associations involving these constructs should therefore be interpreted cautiously. Moreover, because several measures were contextually adapted and some response formats differed from those of the source instruments, the present CFA results establish the differentiation of the adapted constructs within this sample only. They should not be interpreted as evidence that the adapted measures are psychometrically invariant with, or directly equivalent to, the original instrument formats.

All heterotrait–monotrait ratios were below 0.85, with the highest value of 0.760 observed between music learning self-efficacy and enjoyment. Thus, although several constructs were correlated, the HTMT results did not indicate problematic overlap between them.

Harman’s single-factor test showed that the first unrotated factor accounted for 23.93% of the total variance. In addition, the single-factor CFA model reported in Table 3 fit substantially worse than the hypothesized eight-factor model. These limited diagnostics indicated that no single factor dominated the item covariance, although they could not rule out common method bias arising from the single-source, cross-sectional design.

### 4.3 Higher-order structure of music learning engagement

The measurement structure of music learning engagement was examined before the structural model was estimated. A single-factor engagement model demonstrated poor fit, *χ*^2^(54) = 564.20, CFI = 0.773, TLI = 0.722, RMSEA = 0.138, 90% CI [0.127, 0.148], and SRMR = 0.095. In contrast, the correlated three-factor model distinguishing behavioral, cognitive, and agentic engagement demonstrated good fit, *χ*^2^(51) = 110.91, CFI = 0.973, TLI = 0.966, RMSEA = 0.049, 90% CI [0.036, 0.061], and SRMR = 0.039.

Behavioral, cognitive, and agentic engagement were subsequently specified as first-order factors loading on a second-order music learning engagement factor. As reported in Table 5, all three dimensions loaded substantially on the higher-order engagement factor, and all corresponding bootstrap confidence intervals excluded zero.

**Table 5:** Standardized second-order loadings for music learning engagement.

First-order dimension	Standardized loading	95% BC bootstrap CI	*R* ^2^
Behavioral engagement	0.802	[0.731, 0.871]	0.643
Cognitive engagement	0.800	[0.732, 0.862]	0.639
Agentic engagement	0.771	[0.698, 0.838]	0.594

Behavioral, cognitive, and agentic engagement were specified as first-order factors loading on a second-order music learning engagement factor. The higher-order portion of the model was just identified. The 95% bias-corrected bootstrap confidence intervals were obtained from 5,000 case-resampling replications.

Because the higher-order factor was defined by exactly three first-order dimensions, the second-order portion of the model was just identified. The higher-order specification was therefore retained for theoretical and interpretive reasons, not because it demonstrated superior fit relative to the correlated three-factor model.

### 4.4 Full latent structural model

The hypothesized associations were estimated simultaneously in a full latent structural equation model. The disturbances of enjoyment, anxiety, and boredom were allowed to covary because the constructs represented related course-based emotional experiences.

The model produced no negative residual variances or standardized estimates exceeding unity. It demonstrated good fit to the data, *χ*^2^(542) = 812.77, *p* < 0.001, CFI = 0.959, TLI = 0.955, RMSEA = 0.032, 90% CI [0.027, 0.036], and SRMR = 0.042. These indices indicated that the overall structural model was compatible with the observed covariance pattern, although they do not establish the correctness of the directional ordering of the paths.

After teacher autonomy support and self-efficacy were accounted for, the standardized residual association between anxiety and boredom was positive, estimate = 0.478, 95% bootstrap CI [0.371, 0.573]. The standardized residual associations of enjoyment with anxiety, estimate = 0.127, 95% CI [−0.018, 0.271], and boredom, estimate = 0.093, 95% CI [−0.041, 0.239], were not statistically distinguishable from zero.

The standardized structural associations are presented in Table 6 and illustrated in Figure 2. Unless otherwise specified, confidence intervals are 95% bias-corrected bootstrap intervals based on 5,000 replications. Teacher autonomy support was positively associated with music learning self-efficacy [*β* = 0.608, 95% CI (0.527, 0.681)] and negatively associated with anxiety and boredom, whereas its conditional association with enjoyment was not statistically distinguishable from zero. Self-efficacy was positively associated with enjoyment and negatively associated with anxiety and boredom. Enjoyment was positively associated with music learning engagement [*β* = 0.426, 95% CI (0.217, 0.668)], whereas boredom was negatively associated with engagement [*β* = −0.310, 95% CI (−0.463, −0.159)]. Anxiety was not conditionally associated with engagement. Teacher autonomy support retained a small positive association with engagement [*β* = 0.149, 95% CI (0.002, 0.297)], whereas the direct association between self-efficacy and engagement was not statistically distinguishable from zero. The model explained 29.5% of the variance in engagement; *R*^2^ values for the remaining endogenous variables are reported in Table 6.

**Table 6:** Standardized structural associations and explained variance in the full latent structural model.

Panel A. Structural associations			
Structural path	β	Bootstrap SE	95% BC bootstrap CI
Teacher autonomy support → Self-efficacy	0.608	0.039	[0.527, 0.681]
Teacher autonomy support → Enjoyment	−0.050	0.058	[−0.167, 0.061]
Self-efficacy → Enjoyment	0.806	0.052	[0.702, 0.907]
Teacher autonomy support → Anxiety	−0.193	0.070	[−0.327, −0.052]
Self-efficacy → Anxiety	−0.185	0.072	[−0.325, −0.042]
Teacher autonomy support → Boredom	−0.251	0.073	[−0.392, −0.103]
Self-efficacy → Boredom	−0.224	0.077	[−0.377, −0.072]
Teacher autonomy support → Engagement	0.149	0.075	[0.002, 0.297]
Self-efficacy → Engagement	−0.184	0.144	[−0.490, 0.079]
Enjoyment → Engagement	0.426	0.115	[0.217, 0.668]
Anxiety → Engagement	−0.014	0.078	[−0.161, 0.141]
Boredom → Engagement	−0.310	0.078	[−0.463, −0.159]

Panel B. Explained variance		
Endogenous latent variable	*R* ^2^	95% BC bootstrap CI
Music learning self-efficacy	0.369	[0.278, 0.464]
Enjoyment	0.603	[0.512, 0.699]
Anxiety	0.115	[0.060, 0.186]
Boredom	0.182	[0.112, 0.275]
Music learning engagement	0.295	[0.210, 0.421]

*β,* standardized coefficient; BC, bias-corrected. Bootstrap standard errors and 95% bias-corrected bootstrap confidence intervals were based on 5,000 case-resampling replications. All 5,000 bootstrap replications produced admissible finite estimates and were retained for analysis. An association was considered statistically distinguishable from zero when its 95% confidence interval excluded zero.

**Figure 2:**
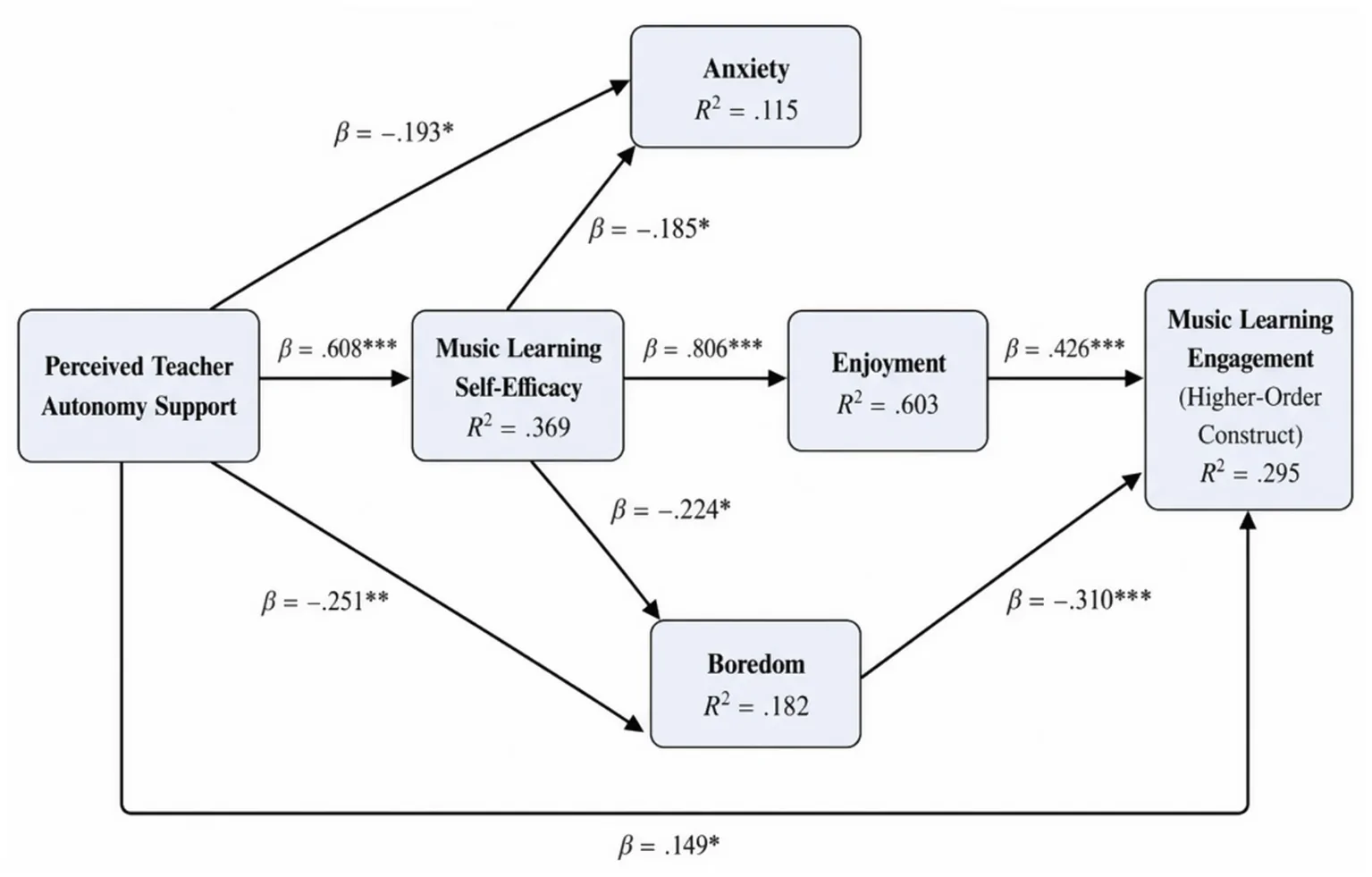
Standardized latent structural model linking teacher autonomy support, music learning self-efficacy, achievement emotions, and music learning engagement. Values on structural paths are standardized coefficients. Values within endogenous constructs are coefficients of determination. Only associations whose 95% bias-corrected bootstrap confidence intervals excluded zero are displayed. The paths from teacher autonomy support to enjoyment (*β* = −0.050), from music learning self-efficacy to engagement (*β* = −0.184), and from anxiety to engagement (*β* = −0.014), whose confidence intervals included zero, are omitted for clarity but are reported in Table 6. The residual covariances among enjoyment, anxiety, and boredom were estimated but are not shown. *p < 0.05; **p < 0.01; ***p < 0.001.

### 4.5 Indirect and total associations

The sequential indirect and total associations are presented in Table 7. Teacher autonomy support showed a positive indirect association with music learning engagement through self-efficacy and enjoyment, β = 0.209, 95% bootstrap CI [0.099, 0.359]. The corresponding pathway through self-efficacy and boredom was also statistically distinguishable from zero, *β* = 0.042, 95% CI [0.011, 0.087]. In contrast, the sequential indirect association through self-efficacy and anxiety was not statistically distinguishable from zero, *β* = 0.002, 95% CI [−0.018, 0.020]. In substantive terms, the bootstrap results indicated that the enjoyment and boredom sequences were reliably different from zero in the specified model, whereas the anxiety sequence was not.

**Table 7:** Sequential indirect and total associations with music learning engagement.

Pathway	Standardized association	Bootstrap SE	95% BC bootstrap CI
Teacher autonomy support → self-efficacy → enjoyment → engagement	0.209	0.065	[0.099, 0.359]
Teacher autonomy support → self-efficacy → anxiety → engagement	0.002	0.009	[−0.018, 0.020]
Teacher autonomy support → self-efficacy → boredom → engagement	0.042	0.020	[0.011, 0.087]
Teacher autonomy support: total indirect association	0.199	0.058	[0.084, 0.311]
Teacher autonomy support: total association	0.349	0.050	[0.247, 0.444]
Self-efficacy: total indirect association	0.415	0.110	[0.228, 0.659]
Self-efficacy: total association	0.231	0.075	[0.081, 0.376]

Bootstrap standard errors and 95% bias-corrected bootstrap confidence intervals were based on 5,000 case-resampling replications. All 5,000 bootstrap replications produced admissible finite estimates and were retained for analysis. Total indirect associations include all indirect pathways implied by the structural model, not only the three sequential pathways displayed in the table. An association was considered statistically distinguishable from zero when its 95% confidence interval excluded zero.

The total indirect association between teacher autonomy support and engagement was positive, *β* = 0.199, 95% CI [0.084, 0.311], and its total association with engagement was *β* = 0.349, 95% CI [0.247, 0.444]. The total indirect association incorporated all indirect pathways implied by the structural model, including both the displayed sequential pathways and shorter indirect pathways through individual mediators. Accordingly, it should not be interpreted as the simple sum of the three sequential pathways shown above. Self-efficacy showed a positive total indirect association with engagement, *β* = 0.415, 95% CI [0.228, 0.659]. Although its direct association with engagement was not statistically distinguishable from zero, its total association with engagement was positive, *β* = 0.231, 95% CI [0.081, 0.376].

The enjoyment pathway had the largest point estimate, although the three sequential indirect associations were not formally compared.

## 5 Discussion

### 5.1 Overview of the findings

This study examined how perceived teacher autonomy support, music learning self-efficacy, and discrete achievement emotions were jointly associated with multidimensional engagement among Chinese undergraduate music students. Taken together, the findings suggest that engagement was related not simply to perceived instructional support or competence beliefs in isolation, but also to the emotional quality of students’ learning experiences. Autonomy support was most clearly associated with stronger competence beliefs and lower negative emotions, whereas engagement itself was more distinctly differentiated by enjoyment and boredom. This pattern underscores the value of considering instructional support, competence beliefs, and discrete emotions together rather than as interchangeable correlates of engagement.

### 5.2 Autonomy support, self-efficacy, and achievement emotions

Perceived teacher autonomy support was positively associated with music learning self-efficacy, consistent with H1. In practical terms, students who perceived their instructors as giving them meaningful choice, acknowledging their perspectives, and supporting initiative also tended to feel more capable of managing the learning and practice demands of their music courses. This pattern is consistent with self-determination theory, which links autonomy-supportive teaching with more adaptive motivational functioning (Reeve and Cheon, 2021; Ryan and Deci, 2020). The association may be particularly relevant in university music courses, where students must meet demanding technical and interpretive standards while developing increasing ownership of practice and artistic decisions. The finding also aligns with research linking autonomy support and social support with academic or musical self-efficacy (Gutiérrez and Tomás, 2019; Bonneville-Roussy and Evans, 2025; Orejudo et al., 2021). Given the cross-sectional design, however, the direction of the association remains uncertain.

Self-efficacy was positively associated with enjoyment and negatively associated with anxiety and boredom, consistent with H2a to H2c. This pattern is broadly consistent with control-value theory, although self-efficacy represents one competence-related belief rather than the complete control appraisal specified by the theory (Pekrun, 2006; Tze et al., 2022). Students who feel more capable of regulating practice, addressing technical difficulties, and making progress may be more likely to experience learning as manageable and rewarding, particularly in relation to enjoyment. This interpretation is consistent with evidence linking positive achievement emotions to adaptive educational outcomes and musical self-efficacy to learning and performance-related functioning (Camacho-Morles et al., 2021; Zelenak, 2024).

Anxiety and boredom, however, warrant separate interpretation. Stronger self-efficacy may be associated with perceiving lessons, evaluation, and corrective feedback as more manageable and less threatening, although anxiety in music education is not limited to formal performance situations (Barros et al., 2022). The association with boredom requires a more conditional interpretation because boredom may arise from low value, insufficient challenge, excessive difficulty, or limited perceived control. These patterns reinforce the value of treating anxiety and boredom as distinct negative emotions.

Teacher autonomy support was negatively associated with anxiety and boredom, consistent with H3b and H3c. Reduced controlling pressure, meaningful rationales, and opportunities for initiative may be consistent with a less threatening learning environment and stronger connections between repetitive technical work and broader musical goals. These processes were not directly measured and should therefore be treated as possible interpretations rather than identified explanations. This interpretation is compatible with evidence that autonomy support can coexist with clear structure and demanding expectations (Patzak and Zhang, 2025; Reeve and Cheon, 2021). In contrast, teacher autonomy support did not show a statistically distinguishable conditional association with enjoyment after accounting for self-efficacy within the full structural model, so H3a was not supported. This pattern is consistent with, but does not establish, the possibility that the association between autonomy support and enjoyment overlaps substantially with competence-related beliefs. Enjoyment may also depend on activity-specific features not represented in the model, including artistic meaning, task difficulty, and opportunities for creative interpretation (Xu and Li, 2025).

### 5.3 Achievement emotions, engagement, and indirect associations

Enjoyment was positively associated with music learning engagement, consistent with H4a, whereas boredom was negatively associated with engagement, consistent with H4b. As a positive activating emotion, enjoyment may correspond with persistence, concentration, strategy use, and active participation. In university music courses, these tendencies may be reflected in sustained practice, constructive use of feedback, and willingness to explore alternative interpretations. Boredom, by contrast, is a negative deactivating emotion associated with attentional withdrawal and reduced effort. It may be especially relevant in music education because sustained individual practice requires students to regulate attention and effort without continuous external monitoring. These findings are consistent with control-value theory and evidence linking achievement emotions with educational outcomes (Camacho-Morles et al., 2021; Pekrun, 2006).

Regarding RQ1, anxiety did not show a statistically distinguishable conditional association with engagement. This may reflect its mixed role as a negative but activating emotion: anxiety can consume cognitive resources and encourage avoidance, but it may also accompany vigilance, preparation, and sustained effort. The null conditional association does not imply that anxiety is unimportant, as it may be more closely related to performance quality, distress, avoidance, or well-being and may vary with intensity, context, and coping resources.

Teacher autonomy support retained a small positive association with engagement after self-efficacy and achievement emotions were included in the model, providing qualified support for H5. This pattern is consistent with broader evidence linking autonomy-supportive or supportive teaching with student engagement (Gutiérrez and Tomás, 2019; Tao et al., 2022). The remaining coefficient may reflect processes not represented in the model, but it does not identify those processes. Because the lower confidence limit was close to zero, replication is needed before this association is treated as stable.

Regarding RQ2, music learning self-efficacy did not retain a statistically distinguishable direct association with engagement after perceived teacher autonomy support and the three achievement emotions were modeled simultaneously. This should not be interpreted as evidence of an inverse association: self-efficacy was positively correlated with engagement at the bivariate level (*r* = 0.273) and showed a positive total association in the structural model [*β* = 0.231, 95% CI (0.081, 0.376)]. The negative but nonsignificant conditional coefficient [*β* = −0.184, 95% CI (−0.490, 0.079)] therefore appears more consistent with substantial overlap among correlated predictors, particularly self-efficacy and enjoyment, than with a substantive negative relationship. In this model, the positive association of self-efficacy with engagement was expressed primarily through its indirect and total associations rather than through a unique direct path.

The theoretically ordered sequential indirect association through self-efficacy and enjoyment was positive and statistically distinguishable from zero, consistent with H6a. The corresponding pathway through self-efficacy and boredom was also statistically distinguishable from zero, consistent with H6b, whereas the anxiety pathway examined under RQ3 was not. Related associations among autonomy-supportive faculty behavior, positive academic emotions, and agentic engagement have been reported in higher education, although the constructs and analytical model were not identical to those used here (Bordbar, 2021). The enjoyment pathway had the largest point estimate, but the indirect estimates were not formally compared. These cross-sectional estimates are consistent with the proposed ordering but do not establish temporal mediation or causal transmission.

### 5.4 Theoretical contributions

The study contributes to research on university music learning by showing that perceived teacher autonomy support, competence-related beliefs, and achievement emotions were not interchangeable correlates of engagement. Perceived autonomy support was associated with self-efficacy, anxiety, boredom, and engagement in different ways, and a small conditional association with engagement remained after the measured psychological variables were included. This pattern extends prior work by showing that the associations of perceived autonomy support with engagement become more differentiated when competence beliefs and discrete achievement emotions are considered simultaneously. At the same time, the findings do not constitute a complete test of self-determination theory or control-value theory because psychological need satisfaction, autonomous motivation, value appraisals, and the full range of control appraisals were not directly measured.

The findings also demonstrate the theoretical importance of distinguishing discrete achievement emotions in music learning. Enjoyment and boredom retained contrasting conditional associations with engagement, whereas anxiety did not. This difference suggests that negative emotional experiences should not be treated as a single category. In particular, the contrast between anxiety as a negative activating emotion and boredom as a negative deactivating emotion may be consequential for understanding sustained attention, effort, and participation during coursework and practice. A general positive-versus-negative emotion framework would have obscured this pattern.

The results further refine the role of music learning self-efficacy by showing that its relation with engagement depends on the level of association being considered. Its positive zero-order and total associations contrasted with the absence of a statistically distinguishable unique direct association once autonomy support and achievement emotions were considered simultaneously. This pattern suggests that competence beliefs should be interpreted in relation to closely connected emotional experiences rather than as isolated predictors of engagement. The higher-order engagement specification additionally provided a parsimonious representation of behavioral, cognitive, and agentic engagement, although its just-identified second-order structure does not establish superiority over the correlated three-factor representation (Reeve and Tseng, 2011; Reeve et al., 2020; Wong and Liem, 2022).

### 5.5 Practical implications

The findings are consistent with the potential value of autonomy-supportive instruction and with attending to students’ competence beliefs and emotional experiences, although they do not provide direct evidence for the effectiveness of any specific instructional approach. In university music teaching, autonomy support may involve meaningful rationales, acknowledgment of students’ perspectives, appropriate opportunities for initiative, and clear expectations communicated without unnecessary coercion (Patzak and Zhang, 2025; Reeve and Cheon, 2021). These practices can be incorporated without reducing technical standards or necessary repetition.

The findings may inform feedback practices that help students recognize attainable progress and connect identified weaknesses with specific next steps. In university music teaching, demanding standards may be more supportive of self-efficacy when feedback clarifies how improvement can be achieved rather than only identifying deficiencies (Usher and Pajares, 2008).

Instructional attention may also extend beyond anxiety to the conditions associated with enjoyment and boredom. Repetitive technical work remains necessary, but instructors may clarify its purpose by connecting exercises with interpretive and performance goals, helping students monitor progress, and providing appropriate opportunities to ask questions or communicate difficulties. These implications remain provisional because the present study did not evaluate an instructional intervention.

### 5.6 Limitations and future research

Several design limitations should be considered. First, the cross-sectional design does not establish temporal or causal ordering, and reciprocal relationships among the study variables remain plausible. The theoretically ordered indirect associations should therefore be interpreted as model-dependent decompositions of covariance rather than evidence of temporal mediation. Second, all focal variables were assessed through student self-report at a single occasion. Although Harman’s single-factor analysis and the single-factor CFA did not indicate dominance by one general factor, common method variance cannot be ruled out. Third, response-quality checks were not prespecified, and completion-time metadata were unavailable. Post-hoc diagnostics did not indicate pervasive problematic responding, but they cannot provide the same protection as prespecified screening procedures.

Measurement limitations also warrant caution. The measures were adapted to university music learning to different degrees, including contextual wording changes, item reduction, and response-format modification. Although the pilot study indicated satisfactory preliminary internal consistency, no formal invariance comparison with the source formats was conducted; the adapted measures therefore should not be assumed to function identically to the original instruments. Convergent validity was also modest for music learning self-efficacy, anxiety, and boredom, with AVE values below 0.50. Although composite reliability, factor loadings, HTMT values, and competing measurement models supported retaining these constructs, structural associations involving them should be interpreted cautiously.

Finally, convenience sampling and non-probability institutional recruitment limit generalizability. Institutional affiliation, course identifiers, and instructor identifiers were not retained at the individual level, preventing reconstruction of the institutional sample distribution and evaluation of potential course- or instructor-level clustering. Because clustering could not be assessed, some confidence intervals may be narrower than they would be under an appropriate multilevel specification. A conventional response rate also could not be calculated because the recruitment denominator was not recorded.

The model also did not assess the full range of control and value appraisals specified by control-value theory or psychological need satisfaction, autonomous motivation, and teacher-provided structure within self-determination theory. Future research should use longitudinal, intensive longitudinal, intervention, and multilevel designs; incorporate multi-source or behavioral measures; retain anonymized institutional and instructional identifiers; and further validate the adapted measures across settings and populations.

## 6 Conclusion

This study showed that perceived teacher autonomy support, music learning self-efficacy, and discrete achievement emotions were differently associated with multidimensional engagement among Chinese undergraduate music students. Enjoyment and boredom retained contrasting conditional associations with engagement, whereas anxiety did not, and self-efficacy showed positive zero-order and total associations without a statistically distinguishable unique direct association. These findings highlight the value of considering autonomy-supportive teaching, competence beliefs, and discrete emotions jointly when examining engagement in university music learning.

## Data Availability

The raw data supporting the conclusions of this article will be made available by the authors, without undue reservation.
