# Supplementary material for: Teacher autonomy support and music learning engagement: conditional associations of self-efficacy and discrete achievement emotions among university music students
Source: Front Psychol. 2026 Sep 29;17:1947710. doi: 10.3389/fpsyg.2026.1947710 (PMC13658468; doi:10.3389/fpsyg.2026.1947710)
Supplement: Supplementary file 1 [file Table_1.DOCX]

Supplementary Table S1. Item-Level Sources and Adaptation Decisions for the Study Measures

Panel A. Perceived Teacher Autonomy Support

| **Code** | **Source construct/content represented** | **Item administered in the present study** | **Adaptation type** | **Rationale** |
| --- | --- | --- | --- | --- |
| **TAS1** | Instructor provides opportunities for choice and self-direction | In this course, the instructor gives me opportunities to choose how I learn or practice. | Contextual wording and referent specification | To anchor perceived choice to the focal music course and to course-related learning or practice. |
| **TAS2** | Student feels understood by the instructor | I feel that this instructor understands me. | Minimal referent modification | To refer consistently to the primary instructor of the focal course. |
| **TAS3** | Instructor communicates confidence in the student’s ability to succeed | This instructor believes that I am capable of doing well in this course. | Minor wording modification | To retain perceived instructor confidence while anchoring success to the focal course. |
| **TAS4** | Instructor encourages student questions | This instructor encourages me to ask questions. | Minimal referent modification | To retain the original autonomy-support content in relation to the focal instructor. |
| **TAS5** | Instructor listens to the student’s preferred way of approaching learning | This instructor listens to how I would like to approach my learning or practice. | Contextual specification | To apply preference acknowledgement specifically to music learning and practice. |
| **TAS6** | Instructor attempts to understand the student’s perspective before suggesting another approach | Before suggesting a new way of learning or practicing, this instructor tries to understand my point of view. | Contextual specification | To situate perspective taking within course learning and practice. |

**Scale-level adaptation.** All six items of the short-form Learning Climate Questionnaire were retained; no item from the six-item short form was omitted. Wording was contextualized to the focal music course and its primary instructor. The source response format was replaced by the common five-point Likert format used in the present study.

**Panel B. Music Learning Self-Efficacy**

| **Code** | **Source construct/content represented** | **Item administered in the present study** | **Adaptation type** | **Rationale** |
| --- | --- | --- | --- | --- |
| **MSE1** | Confidence in successfully learning or mastering musical material | I am confident that I can master the musical knowledge or skills required in this course. | Substantial contextual reconstruction | To operationalize self-efficacy in relation to mastery of knowledge and skills required by the focal music course. |
| **MSE2** | Confidence in managing difficult musical-learning demands | Even when the learning tasks in this course are difficult, I believe that I can complete them. | Substantial contextual reconstruction | To represent perceived capability under difficult but routine course-learning demands. |
| **MSE3** | Confidence in overcoming problems encountered during musical learning or practice | I believe that I can solve the problems I encounter while learning or practicing for this course. | Substantial contextual reconstruction | To focus efficacy judgments on problem solving during ordinary course learning and practice. |
| **MSE4** | Confidence in meeting musical-learning expectations or requirements | I am confident that I can meet the learning requirements of this course. | Substantial contextual reconstruction | To anchor self-efficacy directly to the requirements of the focal university music course. |
| **MSE5** | Confidence in maintaining progress following initial difficulty or ineffective learning | If my learning or practice is not effective at first, I believe that I can adjust my approach and continue to improve. | Substantial contextual reconstruction | To represent perceived capability to adjust strategies and persist following initially ineffective learning or practice. |

**Scale-level adaptation.** The original Self-Efficacy for Musical Learning measure was not reproduced item for item. Five course-focused indicators were developed from content represented in the source construct. The adaptation involved substantial item reduction and contextual reconstruction because the present study concerned routine university course learning and practice rather than preparation for a specific public performance. Content tied primarily to auditions, competitions, concerts, or other formal public-performance situations was not retained. No one-to-one correspondence between the five administered items and five specific source items is claimed. The source response format was replaced by the common five-point format. Accordingly, the present five-item measure should not be interpreted as psychometrically equivalent to the source instrument.

**Panel C. Music Learning Engagement**

**Behavioral Engagement**

| **Code** | **Source construct/content represented** | **Item administered in the present study** | **Adaptation type** | **Rationale** |
| --- | --- | --- | --- | --- |
| **BE1** | Careful listening and attentional focus during instruction | In this course, I listen carefully or pay close attention to the instructor’s guidance. | Content consolidation and contextualization | To represent attentive involvement in relation to the focal instructor’s guidance. |
| **BE2** | Effort and persistence during learning activities | I work hard to complete the learning and practice tasks required in this course. | Contextualization | To represent effort in relation to required music-learning and practice tasks. |
| **BE3** | Careful attention when new material is introduced | When the instructor begins explaining new material, I listen carefully. | Minor contextual wording | To retain attentive involvement while anchoring the item to the focal course. |
| **BE4** | Remaining focused and on task during learning | I stay focused while learning or practicing the content of this course. | Contextual reconstruction | To represent sustained attention across both course learning and music practice. |

**Cognitive Engagement**

| **Code** | **Source construct/content represented** | **Item administered in the present study** | **Adaptation type** | **Rationale** |
| --- | --- | --- | --- | --- |
| **CE1** | Connecting new material with prior knowledge | When learning in this course, I try to connect new material with what I already know. | Contextual wording | To represent elaborative learning in relation to music-course content. |
| **CE2** | Planning what one intends to accomplish before learning | Before I begin learning or practicing, I think about what I need to accomplish. | Contextual wording | To represent prospective planning for course learning and practice. |
| **CE3** | Monitoring understanding or mastery while learning | While learning or practicing, I check how well I understand or have mastered the material. | Contextual wording | To represent metacognitive monitoring during music learning and practice. |
| **CE4** | Changing strategy when the current learning approach is ineffective | If a learning or practice method does not help me understand or master the material, I change my approach. | Contextual wording | To represent strategic adjustment when a learning or practice approach is ineffective. |

**Agentic Engagement**

| **Code** | **Source construct/content represented** | **Item administered in the present study** | **Adaptation type** | **Rationale** |
| --- | --- | --- | --- | --- |
| **AE1** | Asking questions and actively contributing during instruction | In this course, I actively ask questions. | Minor contextual wording | To represent proactive contribution to the instructional process. |
| **AE2** | Communicating learning preferences or desired ways of working | I tell the instructor how I prefer to learn or practice. | Content consolidation and contextual reconstruction | To focus proactive preference communication on music learning and practice. |
| **AE3** | Communicating areas of interest to the instructor | I let the instructor know which learning topics interest me. | Contextual wording | To anchor communicated interests to focal course content. |
| **AE4** | Suggesting ways in which learning activities could be improved | I offer suggestions about how the learning activities in this course could be improved. | Contextual wording | To represent constructive student input into instructional activities. |

Scale-level adaptation. The engagement measure was reduced to balanced four-item representations of behavioral, cognitive, and agentic engagement. Emotional engagement was not administered because positive emotional experience was assessed separately using the learning-related enjoyment measure, thereby reducing conceptual and item-content overlap between engagement and achievement emotion. The selected engagement items were contextualized to the focal music course and related learning or practice and were administered using the common five-point Likert format adopted for the present questionnaire.

**Panel D. Achievement Emotions**

**Learning-Related Enjoyment**

| **Code** | **Source construct/content represented** | **Item administered in the present study** | **Adaptation type** | **Rationale** |
| --- | --- | --- | --- | --- |
| **ENJ1** | Enjoyment of challenging learning material | I enjoy the challenge of learning the content of this course. | Domain contextualization | To anchor challenge-related enjoyment to the focal music course. |
| **ENJ2** | Enjoyment of engaging with learning material | I enjoy learning and working with the content of this course. | Domain contextualization | To refer specifically to learning and working with focal course content. |
| **ENJ3** | Satisfaction with learning progress that supports continued learning | I am pleased with the progress I make in this course, and this makes me want to continue learning. | Domain contextualization | To retain progress-related enjoyment while anchoring it to the focal course. |
| **ENJ4** | Positive excitement when learning is progressing successfully | I feel excited when my learning or practice in this course is going well. | Domain contextualization | To apply positive learning emotion to both course learning and music practice. |

**Learning-Related Anxiety**

| **Code** | **Source construct/content represented** | **Item administered in the present study** | **Adaptation type** | **Rationale** |
| --- | --- | --- | --- | --- |
| **ANX1** | Nervousness and uneasiness during learning | I feel nervous and uneasy while learning in this course. | Domain contextualization and minor wording modification | To assess anxiety specifically during focal-course learning. |
| **ANX2** | Worry about coping with learning demands | I worry about whether I can cope with all the learning or practice tasks in this course. | Domain contextualization | To extend demand-related worry to both course learning and music-practice tasks. |
| **ANX3** | Anxiety during learning accompanied by disruption of attention or concentration | While learning in this course, I sometimes find it difficult to concentrate because I feel anxious. | Substantive wording modification | To represent anxiety-related concentration difficulty within the course-learning context. |
| **ANX4** | Worry about completing learning material accompanied by physiological anxiety | When I worry that I may not be able to complete the learning material in this course, I feel physically tense or uncomfortable. | Substantive wording modification and symptom generalization | To retain the somatic component of learning anxiety while using broader physiological wording appropriate to the course context. |

**Learning-Related Boredom**

| **Code** | **Source construct/content represented** | **Item administered in the present study** | **Adaptation type** | **Rationale** |
| --- | --- | --- | --- | --- |
| **BOR1** | Boredom during learning or study activities | Learning or practicing the content of this course makes me feel bored. | Domain contextualization | To apply learning-related boredom to both course learning and music practice. |
| **BOR2** | Boring learning material leading to mind wandering | The learning content in this course is sometimes so boring that my mind wanders. | Domain contextualization and minor wording modification | To represent attentional drift caused by boring focal-course content. |
| **BOR3** | Desire to postpone boring learning tasks | Because the learning or practice tasks are boring, I sometimes want to postpone them until later. | Domain contextualization | To apply boredom-related postponement to course learning and practice tasks. |
| **BOR4** | Difficulty maintaining concentration because learning is boring | While learning or practicing the content of this course, I sometimes find it difficult to concentrate because I am bored. | Domain contextualization and minor wording modification | To represent boredom-related concentration difficulty across learning and practice. |

**Scale-level adaptation.** All four learning-related items were retained for each of enjoyment, anxiety, and boredom. Most modifications involved contextualizing the wording and instructions to the focal music course and related learning or practice, although two anxiety items involved broader substantive wording changes concerning concentration difficulty and physiological tension. The enjoyment and anxiety instructions explicitly clarified that responses referred to course learning and practice rather than formal stage performances, competitions, or performances in front of an audience; the boredom instructions referred generally to learning and practice in the focal course. The original five-point AEQ-S response format was retained.

**Note.** TAS = perceived teacher autonomy support; MSE = music learning self-efficacy; BE = behavioral engagement; CE = cognitive engagement; AE = agentic engagement; ENJ = learning-related enjoyment; ANX = learning-related anxiety; BOR = learning-related boredom. Source construct/item content is summarized rather than reproduced verbatim. The “Item administered in the present study” column presents an English rendering corresponding to the Chinese items administered in the study questionnaire. For music learning self-efficacy, no one-to-one correspondence between the five administered items and five specific source items is claimed because the adaptation involved substantial item reduction and contextual reconstruction. The response format was standardized to a five-point Likert scale ranging from 1 (strongly disagree) to 5 (strongly agree). The original seven-point response formats of the Learning Climate Questionnaire and the Self-Efficacy for Musical Learning measure were replaced by the common five-point format used in the present study. The engagement items were likewise administered using the common five-point format adopted for the present questionnaire, whereas the original five-point AEQ-S response format was retained.
